# Supplementary material for: Interface-mediated Kirkendall effect and nanoscale void migration in bimetallic nanoparticles during interdiffusion
Source: Nat Commun. 2019 Jun 27;10:2831. doi: 10.1038/s41467-019-10623-0 (PMC6597554; doi:10.1038/s41467-019-10623-0)
Supplement: Supplementary file 1 — Supplementary Information [file 41467_2019_10623_MOESM1_ESM.pdf]

Supplementary Information for

Interface Mediated Kirkendall Effect and Nanoscale Void Migration in Bimetallic Nanoparticles During Interdiffusion

See Wee Chee[1, 2], Zicong Marvin Wong[3], Zhaslan Baraissov[1, 2], Shu Fen Tan[1, 2], Teck Leong Tan[4], and Utkur Mirsaidov[1, 2, 5, 6 *]

1. Department of Physics, National University of Singapore, Singapore 117551
2. Centre for BioImaging Sciences, Department of Biological Sciences, National University of Singapore, Singapore 117557
3. Department of Chemistry, National University of Singapore, Singapore 117543
4. Institute of High Performance Computing, Agency for Science, Technology and Research, Singapore 138632
5. Centre for Advanced 2D Materials and Graphene Research Centre, National University of Singapore, Singapore 117546
6. Department of Materials Science and Engineering, National University of Singapore, Singapore 117575

* Corresponding author: mirsaidov@nus.edu.sg

Supplementary Notes

Details about the As-synthesized Nanoparticles

Low magnification images of the nanoparticles (NPs) after drop casting on the heating chips are provided as Supplementary Figure 1. In general, the NPs tend to cluster after drying and there are a small number of larger irregularly shaped NPs mixed together with the synthesized cuboidal NPs.

For the as-synthesized Au–Pd core–shell NPs (Supplementary Figure 1a), the Au cuboid cores are 27 ± 4 nm in size and the total size including the shell is 49 ± 5 nm. We also mention here that these samples include a significant number of Pd nanocubes because the Pd shell deposition recipe is similar to that for synthesizing Pd nanocubes. As such, when we performed the subsequent steps to generate the sandwich NPs, both Au–Pd–Au NPs and Pd–Au core–shell NPs are present in the samples (Supplementary Figure 1c). The sandwich Au–Pd–Au NPs are 69 ± 8 nm in size.

As shown in Supplementary Figure 1b, the Pd–Au core–shell NP synthesis produces mostly Pd–Au NPs. However, the NPs show up in different orientations because of the non-cuboid shape of the NPs after shell deposition and not all the NPs will present a clear cuboid core. The NPs selected for our studies have an average size of the Pd cores is 22 ± 4 nm and the total size of the Pd–Au NPs is 43 ± 3 nm.

In the scanning transmission electron microscopy (STEM) and energy dispersive X-ray spectroscopy (EDX) experiments, we only collected the images and maps from isolated NPs or from NPs with limited contact with other NPs.

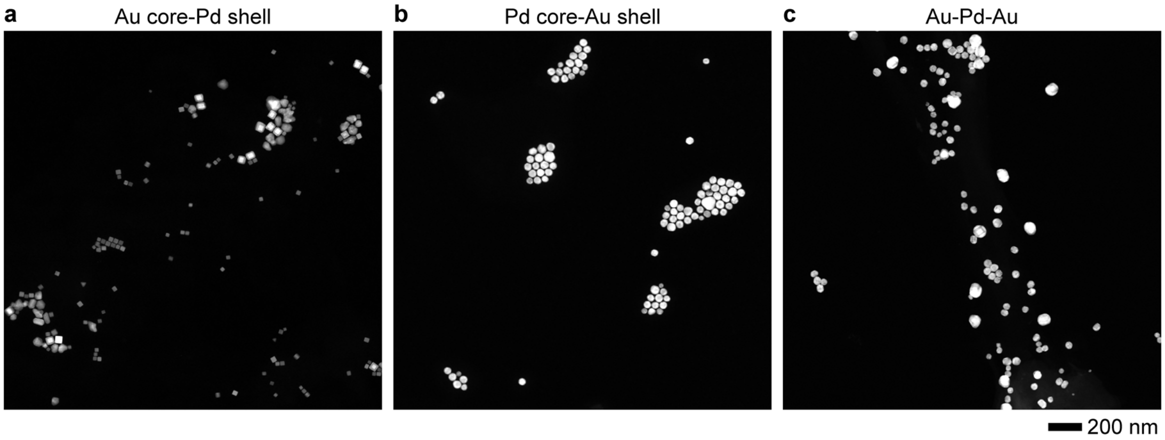

Supplementary Figure 1. Low magnification STEM images of the as-synthesized NPs.

Supplementary Methods

Extracting Diffusion Coefficients from the EDX Elemental Maps

To obtain the diffusion coefficients for Au diffusing into Pd (Supplementary Figure 2) and Pd diffusing into Au (Supplementary Figure 8), we performed a series of experiments where the NPs were heated to one temperature and held there for different time intervals. Supplementary Figure 3 shows a representative heating profile where the NPs were heated to 500 °C for intervals of 1 min, 1 min (accumulated duration: 2 min) and 3 min (accumulated duration: 5 min). EDX maps were collected after lowering the temperature back to 23 °C. Typically, we collected maps of 3 to 4 NPs for each temperature and the maps were acquired until the NPs become random alloys. Next, these EDX maps were processed to extract diffusion profiles of the cores.

Our image processing algorithm was implemented in Python 2.7 and utilized libraries such as OpenCV,[1] Numpy,[2] Scipy,[3] Scikit-image,[4] Pandas.[5] First, we applied a Gaussian filter with σ = 5 pixels to the elemental maps to remove high-frequency noise. The smoothened images were then converted to binary images by applying global Otsu thresholding.[6] To find the orientation of the particles, we extracted the NP outline in the binary image at t = 0 min of each temperature series (Supplementary Figure 2 and 8) and fitted a minimum area rectangle using OpenCV contours class. Then, the centroid position of each NP was found as the mean coordinate of the binary object. We next rotated and shifted the grayscale EDX images such that each NP was positioned at the center of the frame and the NP's edges were parallel to rows and columns of the image. The images at different heating times were also normalized to have the same total pixel intensities. Finally, two sets (along x and x) of elemental profiles were obtained by calculating the average intensity along 25-pixel-wide rectangle, where the width would correspond to 10 – 15 % of the size of the particle. The profiles from different heating times were fitted to a two-sided error function as given below to extract the diffusion coefficient D.

$$C_{core} = \frac{C_0}{2}\left[\mathrm{erf}\left(\frac{x+\Delta x/2}{\sqrt{4Dt}}\right) - \mathrm{erf}\left(\frac{x-\Delta x/2}{\sqrt{4Dt}}\right)\right] \quad (1)$$

Hence, one NP provides two measurements of D at each heating time. Next, we calculate an average diffusion coefficient from all the NPs and heating times. The activation energies for diffusion were obtained from a fit of the average diffusion coefficients at each temperature to $1/T$ where T is the temperature in Kelvin.

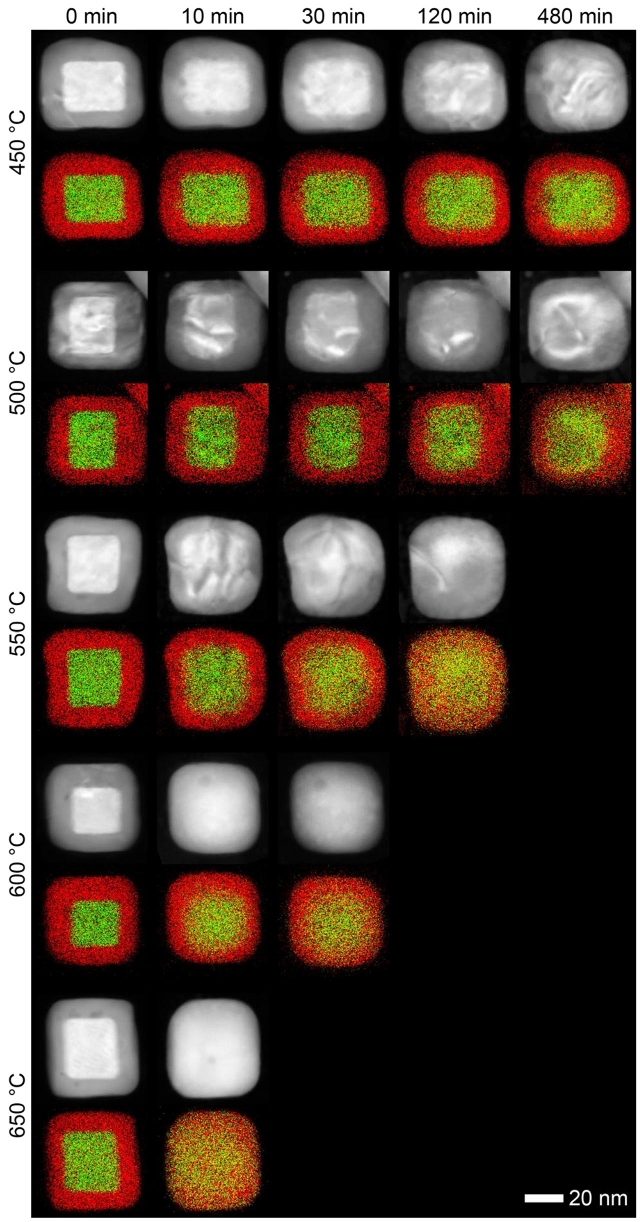

Supplementary Figure 2. Select STEM images and their corresponding EDX maps of Au–Pd core–shell NPs that were tracked as a function of temperature and time. Green denotes Au and red denotes Pd. The zero minute images and maps were collected at 23 °C.

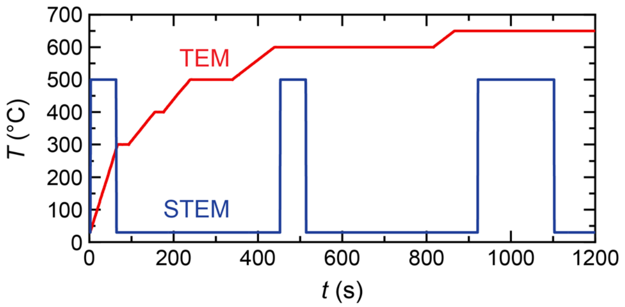

Supplementary Figure 3. Representative heating profile for the TEM (*red*) and STEM (*blue*) experiments.

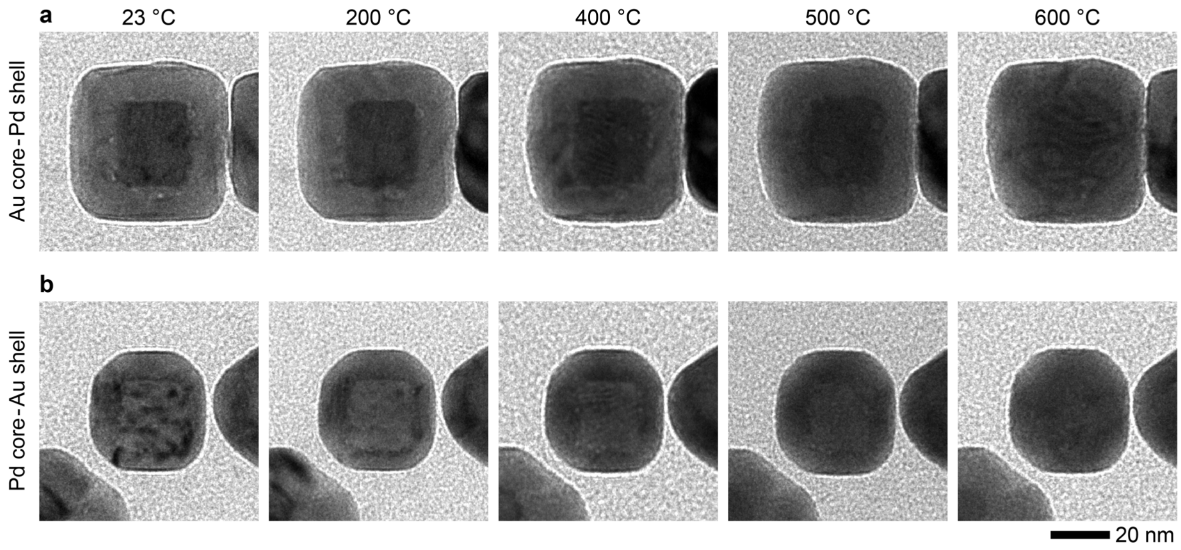

Supplementary Figure 4. TEM image sequence of the morphological evolution of **(a)** Au core–Pd shell and **(b)** Pd core–Au shell NPs as they are heated from 23 °C to 600 °C.

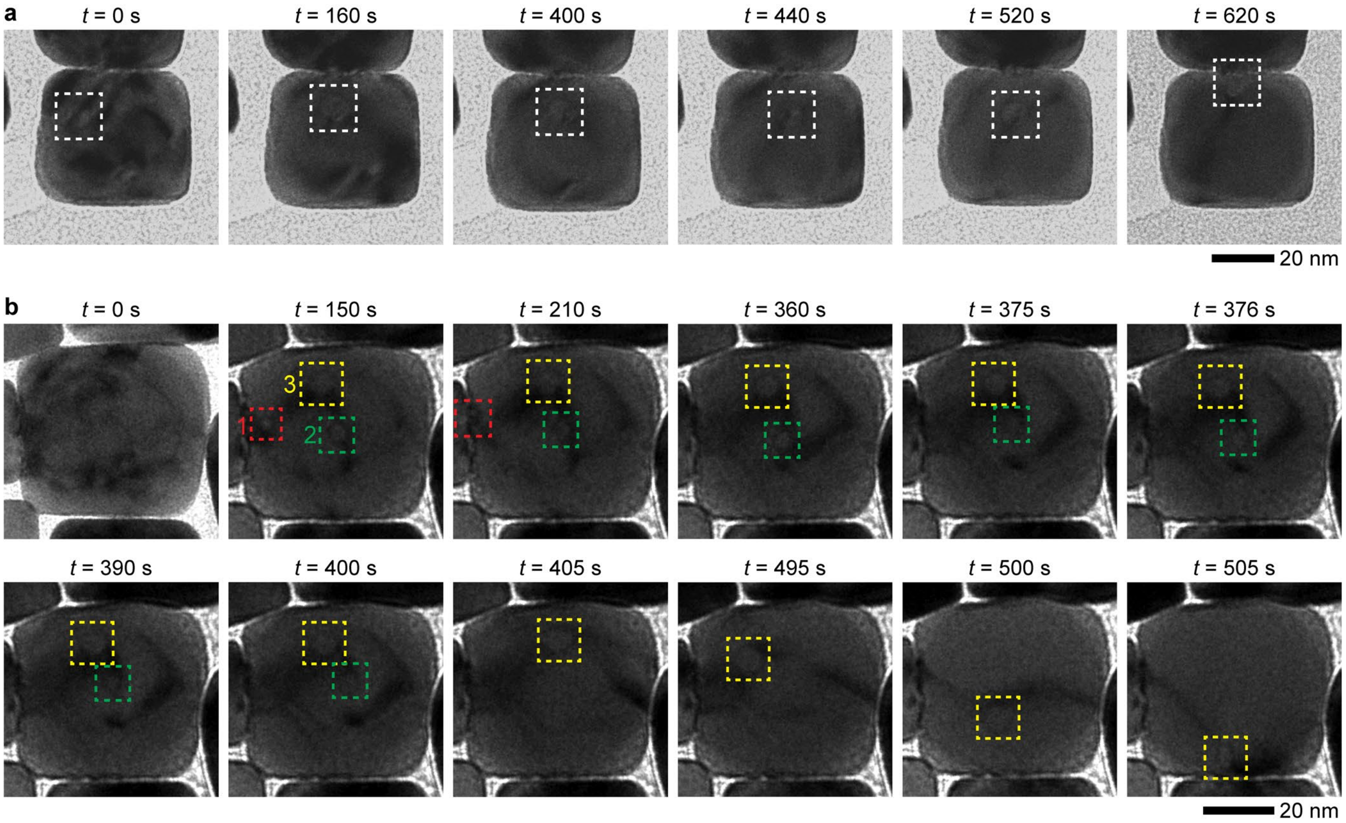

Supplementary Figure 5. (a) TEM image sequence of another Au core–Pd shell NP heated to 650 °C where we observed dislocation pinning by the void highlighted with the white dashed box (Supplementary Movie 2). The band of dark diffraction contrast (t = 400 – 520 s) indicates the presence of a dislocation that is pinned by the void. More importantly, we see here that the motion of the void is associated with the motion of the dislocation. The void is annihilated at the surface at t = 620 s. **(b)** TEM image sequence of a Au core–Pd shell NP heated to 550 °C where three voids (denoted as 1-3 and highlighted with dashed boxes) appear to be pinning a single dislocation (Supplementary Movie 3). The sequence follows the void dynamics as the dislocation straightens. Between t = 150 – 400 s, the position of void 2 shifts more frequently than illustrated by the selected frames provided.

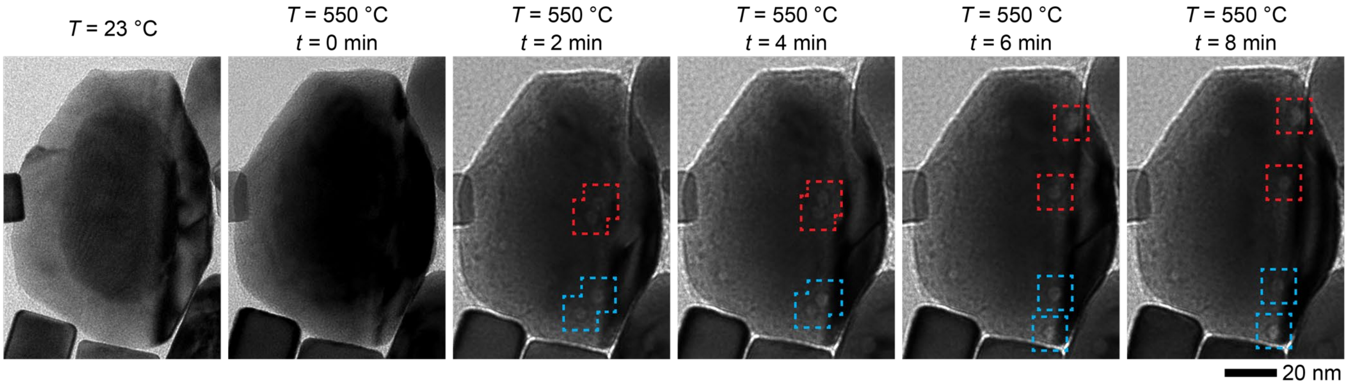

Supplementary Figure 6. TEM image sequence depicting void formation and migration in an irregularly shaped Au core–Pd shell NP that is found together with the cuboid Au core–Pd shell NPs during heating to 550 °C. The dashes boxes highlight four voids and their associated motion.

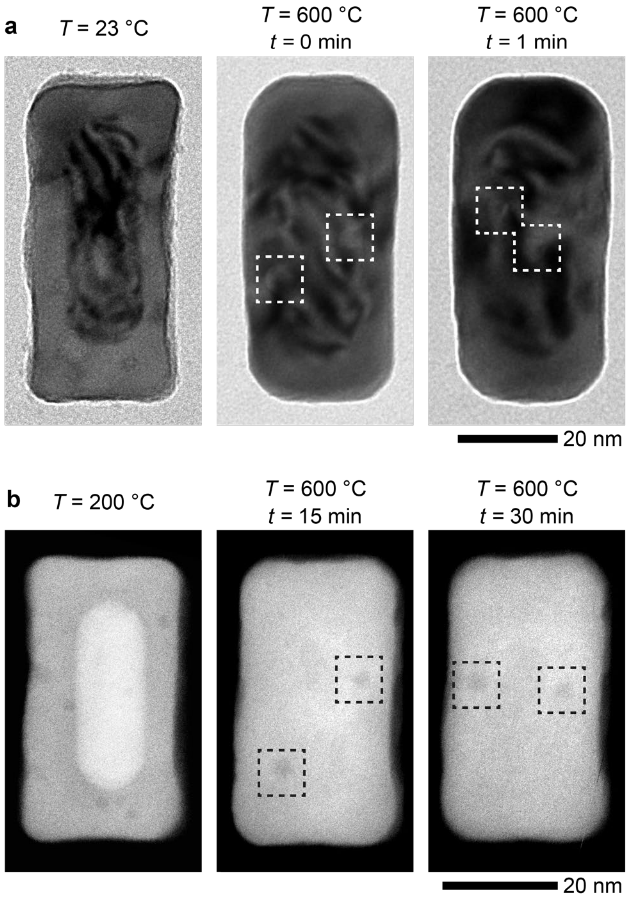

Supplementary Figure 7. (a) TEM and **(b)** STEM image sequences from experiments where we heated Au core–Pd shell NPs with Au nanorod cores.[7] Again, void formation and migration can be observed in in these NPs. These NPs had to be heated first to ~200 °C to reduce contamination from the residual material that was left on the chip surface after drop casting. Dashed boxes highlight voids in the images.

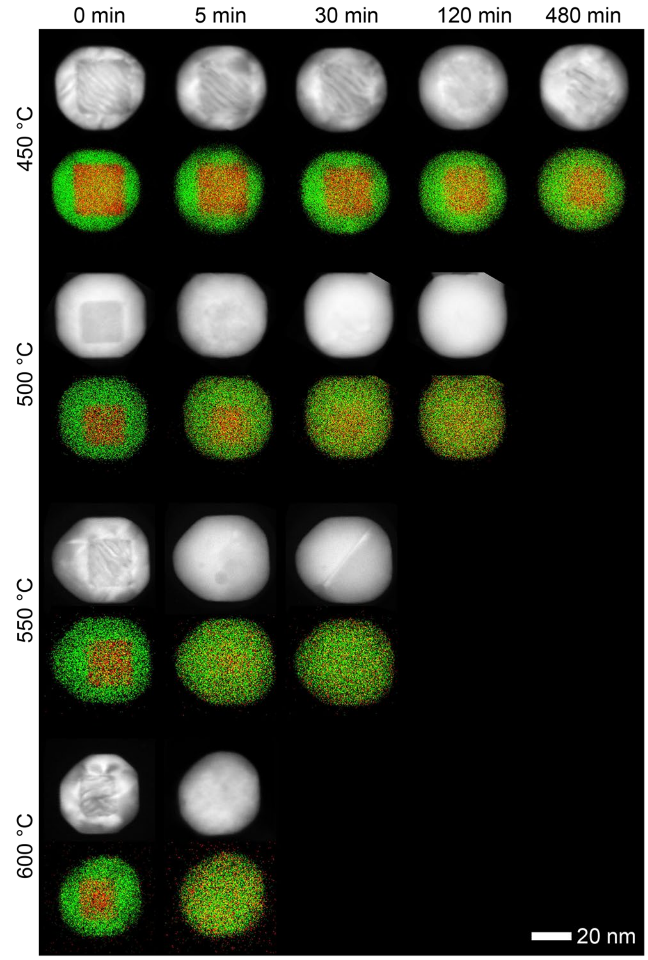

Supplementary Figure 8. Select STEM images and their corresponding EDX maps of Pd core–Au shell NPs that were tracked as a function of temperature and time. Green denotes Au and red denotes Pd. The zero minute images and maps were collected at 23 °C.

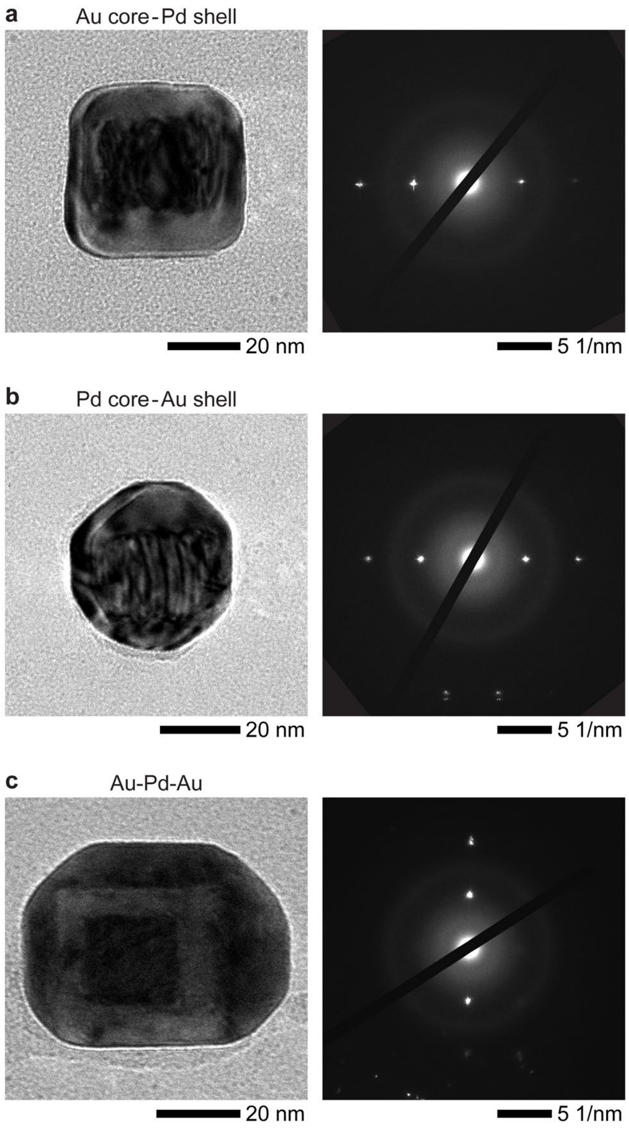

Supplementary Figure 9. TEM images of individual NPs and their corresponding electron diffractions. The Bragg spots indicate that the shells grow in an epitaxial manner on top of the cores, maintaining coherence between the core and shell.

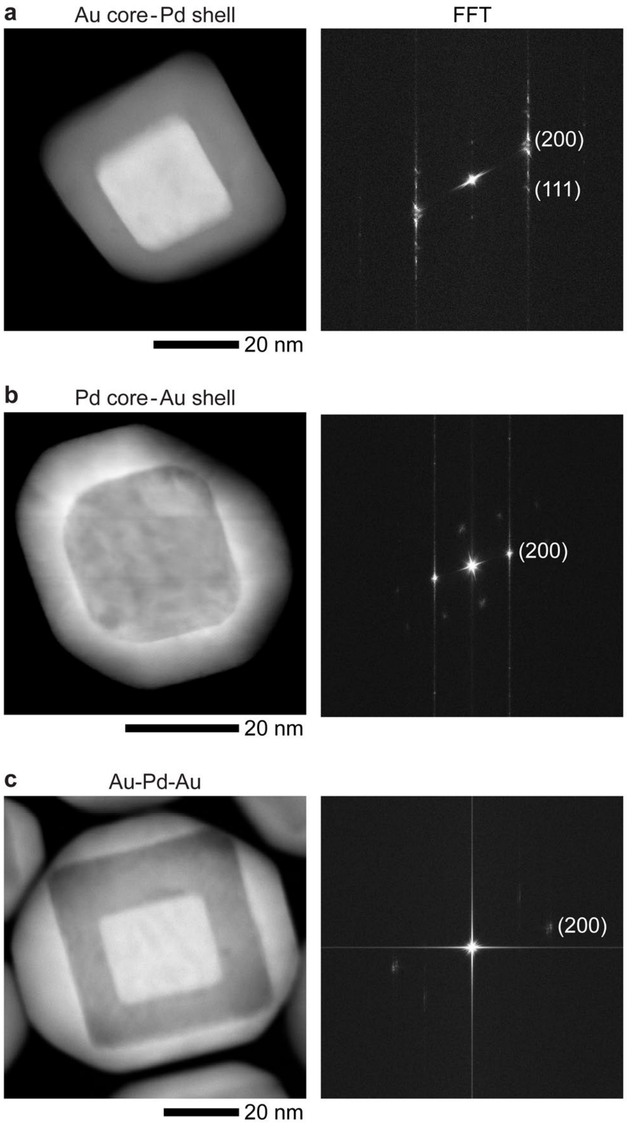

Supplementary Figure 10. STEM images of individual NPs and their corresponding Fourier transforms. These images also indicate that the interface is coherent.

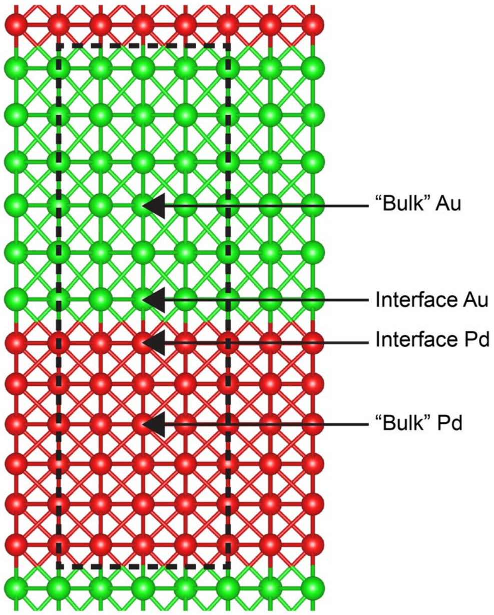

Supplementary Figure 11. Schematic representation of the supercell model utilized for the DFT calculations used in this work. The green and red spheres correspond to Au and Pd, respectively. The unit cell is illustrated by the black dashed lines, comprising of 6 layers of Au atoms stacked onto 6 layers of Pd atoms in the (100) direction.

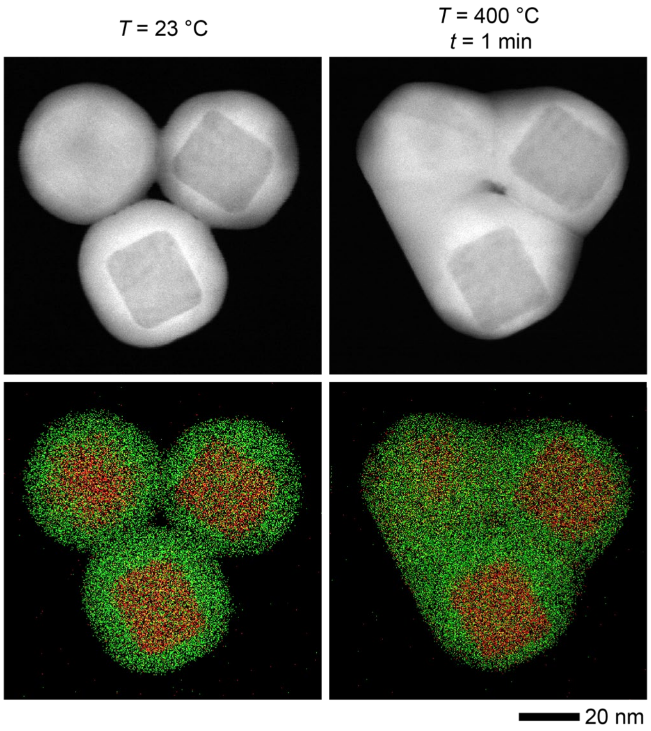

Supplementary Figure 12. STEM and EDX mapping images of a cluster of three Pd core–Au shell NPs that were plasma cleaned prior to heating. It can be seen here that even a moderate heat treatment of 400 °C and 1 min led to significant surface restructuring and NP agglomeration. We attribute these changes to the removal of surface cetyltrimethylammonium bromide surfactant molecules by plasma cleaning. Conversely, morphology of the cores did not appear to change significantly.

	Vac. formation energies (eV)	
	Au core–Pd shell	Pd core–Au shell
**Au Vacancies**		
Au Vacancy in Au bulk	0.38	0.44
Au Vacancy at Au-side of Interface	0.96	0.86
**Pd Vacancies**		
Pd Vacancy in Pd bulk	1.25	1.38
Pd Vacancy at Pd-side of interface	1.19	1.10
Pd Vacancy crosses the bimetallic interface, moving one Au atom across the interface into Pd	0.70	0.64
Pd Vacancy in Au bulk, with the Au atom remaining at the Au–Pd interface	0.22	0.31
Pd Vacancy in Au bulk, with the Au atom diffusing into Pd bulk	0.13	0.09

Supplementary Table 1. Formation energies of Au and Pd vacancies at a bimetallic Au–Pd interface obtained from the first principles density functional theory calculations. Note that these calculations indicate that the lowest energy state is for Au atoms to diffuse into Pd and Pd vacancies to diffuse into Au.

Supplementary References

1. Bradski, G. & Kaehler, A. *Learning OpenCV: Computer Vision with the OpenCV Library. O'Reilly Media* (O'Reilly Media, 2008). doi:10.1109/MRA.2009.933612
2. Van Der Walt, S., Colbert, S. C. & Varoquaux, G. The NumPy array: A structure for efficient numerical computation. *Comput. Sci. Eng.* **13,** 22–30 (2011).
3. Millman, K. J. & Aivazis, M. Python for scientists and engineers. *Computing in Science and Engineering* **13,** 9–12 (2011).
4. van der Walt, S. *et al.* scikit-image: image processing in Python. *PeerJ* **2,** e453 (2014).
5. McKinney, W. Data Structures for Statistical Computing in Python. in *Proceedings of the 9th Python in Science Conference (SCIPY 2010)* 51–56 (2010). doi:10.1016/S0168-0102(02)00204-3
6. Otsu, N. A threshold selection method from gray-level histograms. *IEEE Trans. Syst. Man. Cybern.* **9,** 62–66 (1979).
7. Tan, S. F. *et al.* In Situ Kinetic and Thermodynamic Growth Control of Au-Pd Core-Shell Nanoparticles. *J. Am. Chem. Soc.* **140,** 11680–11685 (2018).
