# Supplementary material for: Interface-mediated Kirkendall effect and nanoscale void migration in bimetallic nanoparticles during interdiffusion
Source: Nat Commun. 2019 Jun 27;10:2831. doi: 10.1038/s41467-019-10623-0 (PMC6597554; doi:10.1038/s41467-019-10623-0)
Supplement: Supplementary file 3 — Description of Additional Supplementary Files [file 41467_2019_10623_MOESM3_ESM.pdf]

Description of Additional Supplementary Files

File Name: Supplementary Movie 1
Description: Void migration in a Au-core, Pd-shell nanoparticle at 650 °C (depicted in Figure 1). The movie has been accelerated by 30×.

File Name: Supplementary Movie 2
Description: Void migration in another Au core–Pd shell nanoparticle at 650 °C. Here, the nanoparticle is oriented such that the dislocation driving void motion shows up with clear (dark) diffraction contrast. The movie has been accelerated by 30×

File Name: Supplementary Movie 3
Description: Void migration in another Au core–Pd shell nanoparticle at 550 °C. In this nanoparticle, we see what appears to be three voids pinning a single dislocation and their subsequent annihilation. The movie has been accelerated by 30×.

File Name: Supplementary Movie 4
Description: Void migration in a Au–Pd–Au sandwich nanoparticle at 600 °C (depicted in Figure 4). The movie has been accelerated by 30×

File Name: Supplementary Movie 5
Description: Void migration in a Au–Pd–Au sandwich nanoparticle at 650 °C (depicted in Figure 4). The movie has been accelerated by 30×.
